# Chronic Exposure to Bioaerosols in PM2.5 from Garbage Stations Accelerates Vascular Aging via the NF‐κB/NLRP3 Pathway

**DOI:** 10.1002/advs.202404142

**Published:** 2024-10-22

**Authors:** Peier Chen, Xiaodong Ning, Weijing Feng, Yajing Li, Guoqin Chen, Xu Shi, YuXuan Pan, Xueqin Shi, Yafang Xiao, Yuhua Liu, Guoxia Zhang, Feiran Zhou, Caiwen Ou

**Affiliations:** ^1^ The Tenth Affiliated Hospital (Dongguan People's Hospital) The First School of Clinical Medicine Southern Medical University Dongguan 523059 China; ^2^ Department of Cardiology State Key Laboratory of Organ Failure Research Guangdong Provincial Key Laboratory of Cardiac Function and Microcirculation Nanfang Hospital Southern Medical University Guangzhou 510515 China; ^3^ Department of Cardiology, The Affiliated Panyu Central Hospital of Guangzhou Medical University Guangzhou 511400 China; ^4^ Department of General Practice The Tenth Affiliated Hospital (Dongguan People's Hospital) Southern Medical University Dongguan 523059 China; ^5^ Department of Environmental Health, Guangdong Provincial Key Laboratory of Tropical Disease Research, School of Public Health Southern Medical University Guangzhou 510515 China; ^6^ Department of Cardiology The First Hospital of Hunan University of Chinese Medicine Changsha 410007 China

**Keywords:** bioaerosol, garbage station, NF‐κB/NLRP3, PM2.5, vascular aging

## Abstract

The fine particulate matter (PM2.5) in air pollution is a critical risk factor influencing human health. Our study included 8144 participants and showed that the risk of major adverse cardiovascular events increases by 35% (HR, 1.35; 95% CI, 1.14–1.60) for participants with the highest quartile to PM2.5 exposure as compared to those with lowest quartile. Bioaerosols, as an important environmental exposure in PM2.5, can induce systemic chronic inflammation leading to vascular aging. Thus, the effects of bioaerosols are investigated from household garbage stations in PM2.5 on vascular aging, and the underlying mechanisms are explored. In vivo, chronic exposure to bioaerosols upregulated senescence marker expression levels while causing vascular dysfunction and remodeling. In vitro, bioaerosol exposure induced decreased proliferation, G0/G1 arrest, and impaired migration of human umbilical vein endothelial cells (HUVECs). Furthermore, a single bacterium (AS22a) from the bioaerosol community was isolated and demonstrated that it upregulated inflammatory factors and accelerated cell senescence and vascular aging by activating the NF‐κB/NLRP3 signaling pathway, which may serve as a primary mechanism underlying vascular aging induced by bioaerosols in PM2.5. These findings suggest that high levels of bioaerosols in household garbage stations may adversely affect cardiovascular health.

## Graphical abstract

These findings indicate that the high concentrations of conditionally pathogenic bioaerosols in PM2.5 from garbage stations, may accelerate vascular aging. AS22a, as a bacterial component of bioaerosols, can stimulate vascular endothelial senescence via the NF‐κB/NLRP3 signaling pathway. This study first proves that bioaerosols in PM2.5 can accelerate vascular aging via the NF‐κB/NLRP3 signaling pathway.

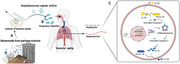

## 1 Introduction

PM2.5 is harmful to human health and the atmosphere due to its small particle size, high concentration of toxic substances, long atmospheric residence time, and ability to travel long distances, facilitating its entry into the human circulatory system. In recent decades, increasing epidemiological evidence has linked short‐term exposure to air pollutants with acute coronary syndrome (ACS)‐related events. The findings of time‐stratified case‐crossover studies have consistently demonstrated a significant association between both acute and chronic exposure to PM2.5 and the incidence, initial hospitalization, recurrent hospitalization risk, as well as premature mortality from ACS and its subtypes.^[^ ^1^ ^]^ Bioaerosols are typically described as a multiphase system with biological components such as viruses, bacteria, and fungal spores in the air,^[^ ^2^ ^]^ which can induce inflammation and alter the human microbiome, has a significant impact on the immune system and overall health of individuals. The intersection of bioaerosols with human health, the environment, and the microbiome necessitates further investigation into the effects and mechanisms of bioaerosol exposure in PM2.5 on the development of cardiovascular disease (CVD), which holds significant research value.

As a place for daily collection and treatment of various waste, garbage stations are full of harmful bioaerosol‐containing pathogenic and opportunistic pathogens, posing a significant threat to human health.^[^ ^3^ ^]^ Numerous studies have indicated that prolonged exposure to garbage cleaning personnel can lead to imbalances in respiratory tract flora, weakened immunity, and increased disease risk.^[^ ^4^ ^]^ The incidence of hypertension and coronary heart disease has significantly risen among populations residing near garbage stations.^[^ ^5^ ^]^ Studies have demonstrated that long‐term exposure to air pollution may be a causal link between biological aging and cardiovascular disease.^[^ ^6^ ^]^ Additionally, a high concentration of bioaerosols in the blood has been found to accelerate the physiological aging process of the body.^[^ ^7^ ^]^ However, the underlying mechanism of bioaerosol‐related vascular aging remains unknown.

Previous studies^[^ ^8^ ^]^ have confirmed that air pollutants can cause endothelial dysfunction, stimulate the release of inflammatory factors, and trigger a systemic inflammatory response. Inflammation is considered an endogenous factor of aging, which promotes aging and releases a large number of inflammatory factors such as IL‐6, TNF‐α, IL‐1β, etc. NF‐κB is an essential factor in the transcription of inflammatory factors. The activity of NF‐κB is significantly increased in senescent endothelial cells. Inhibition of NF‐κB activity can effectively alleviate or delay cell senescence.^[^ ^9^ ^]^ In many age‐related diseases, such as atherosclerosis and diabetes, NF‐κB is continuously activated.^[^ ^10^ ^]^ As a key mediator in the inflammasome family, NOD‐like receptor protein 3 (NLRP3) can be activated by a series of danger signals such as metabolic wastes, stress stimuli, and internal and external harmful metabolites. Subsequently triggered activation of downstream genes ultimately leads to the induction of an inflammatory response.^[^ ^11^ ^]^ Additionally, experiments have shown that the expression level of NLRP3 inflammasome in the large arteries of aged mice is significantly increased, with its expression level also increasing with age, indicating that the NLRP3 inflammasome plays an important role in aging.^[^ ^12^ ^]^ We speculate that bioaerosols from garbage stations may be recognized by NLRP3 inflammasome after being inhaled by the lung into the systemic circulation, mediating the synthesis and release of inflammatory factors as well as the recruitment of inflammatory cells, and participating in the senescence process of vascular endothelial cells.

**Scheme 1:**
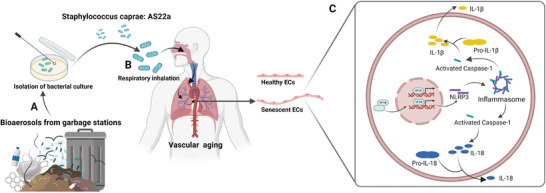
Bioaerosols from garbage stations accelerated vascular endothelial senescence. A) Garbage station with a high concentration of bioaerosols. B) Staphylococcus caprae AS22a was inhaled by the lung into the systemic circulation‐induced vascular aging. C) Bioaerosols accelerated vascular endothelial senescence through the NF‐κB/NLRP3 signaling pathway.

Air pollution is closely associated with inflammatory response, leading to endothelial dysfunction and vascular aging.^[^ ^13^ ^]^ We hypothesized that bioaerosols from garbage stations might exert an impact on vascular aging. Therefore, this study aimed to examine the effects and underlying mechanisms of bioaerosols from garbage stations on vascular aging (**Scheme** **1**). First, we collected bioaerosols from the garbage environment and evaluated their potential health hazards. Next, we explored the effects of bioaerosols from garbage stations on vascular aging at both the animal and cellular levels. Subsequently, a strain of Staphylococcus caprae AS22a was isolated and identified from the community of bioaerosols, and explored the effects of AS22a on vascular endothelial cell senescence and its potential molecular mechanisms from an inflammatory perspective. This study will reveal the toxic effects of chronic exposure to high concentrations of harmful bioaerosols on vascular endothelial cell senescence and provide a reference for evaluating occupational exposure risks in certain special industries.

## 2 Results

### 2.1 PM2.5 Increased Cardiovascular Outcomes Incidence in a Dose‐Dependent Manner

**Figure 1:**
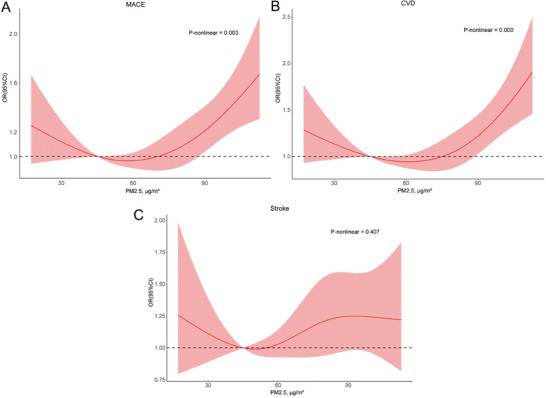
Dose‐response functions of PM2.5 on cardiovascular outcomes incidence within the multivariate‐adjusted models. A) Major adverse cardiovascular event (MACE) incidence. B) Cardiovascular disease (CVD). C) Stroke. Exposure was fitted as a smooth term using a restricted cubic spline with 4 knots. In high levels of PM2.5 exposure, the dose‐response function slope for MACE and CVD is steeper. MACE, major adverse cardiovascular event; CVD, cardiovascular disease; OR, odds ratio; CI, confidence interval. Adjusted for age, gender, education, residence, body mass index, smoking status, alcohol consumption, hypertension status, diabetes status, dyslipidemia status, kidney disease status at baseline.

This study included 8144 participants with an average PM2.5 concentration of 65.9 ± 23.6 µg m^−3^ (Table S1, Supporting Information). Comparing the highest quartile to the lowest quartile, the ORs were 1.35 (95% CI, 1.14‐1.60) for incident MACE, 1.48 (95% CI, 1.22–1.78) for incident CVD, and 1.11(95% CI, 0.85–1.45) for incident stroke (Table S2, Supporting Information). The results remained statistically significant even after fully adjusted by age, gender, education, residence, body mass index, smoking status, alcohol consumption, hypertension status, diabetes status, dyslipidemia status, and kidney disease status at baseline. The RCS curves of MACE and CVD incidence showed a similar upward trend. The association between PM2.5 exposure and both MACE and CVD is non‐linear across the entire range of exposure (**Figure** **1**). The curves exhibit steeper slopes at higher levels of PM2.5 exposure (approximately greater than 85 µg m^−3^).

### 2.2 Diversity Analysis of Bioaerosols from Garbage Stations

**Figure 2:**
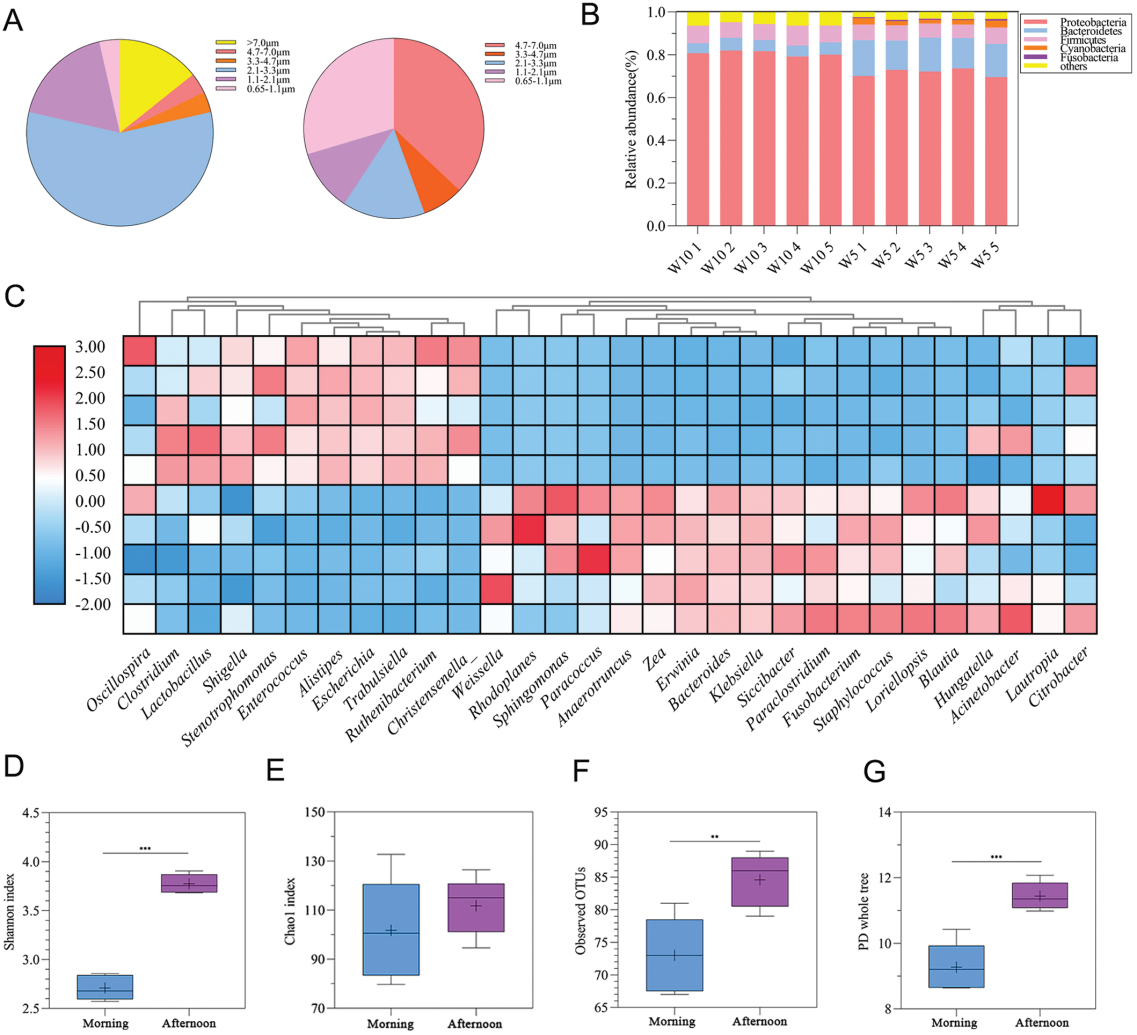
Analysis of bioaerosols from garbage stations. A) The aerodynamic diameter of bioaerosols from garbage stations during two time periods. B) Relative abundance of bacteria at the phylum classification level during two time periods. C) Relative abundance of bacteria at the genus classification level during two time periods. D‐G) Comparison of the diversity of bacterial flora in bacterial alpha diversity analysis between two time periods.

First, we selected a household garbage station in Guangzhou, China, to collect bioaerosols during two time periods, morning (9:00–12:00 am) and afternoon (2:00–5:00 pm), and conducted bacterial composition testing. The bacterial concentrations of bioaerosols from the garbage station during the two time periods were 224 and 749 CFU m^−3^ (Table S3, Supporting Information), respectively. Compared to the bioaerosols concentrations reported in previous studies on garbage stations in office buildings and schools,^[^ ^14^ ^]^ household garbage stations exhibit higher concentrations. This suggested a potential health risk to the surrounding population near household garbage stations. Moreover, it was observed that the bacterial concentration of bioaerosols sampled in the afternoon was significantly higher than in the morning, suggesting that this difference may be primarily attributed to the operational patterns of the household garbage station affecting the occurrence of bioaerosols. Subsequently, we analyzed the aerodynamic diameter of the collected bioaerosols. As shown in **Figure** **2**A, bioaerosols with a particle size smaller than 3.33 µm accounted for over 75% in the morning and over 55% in the afternoon. Based on the 16S rRNA sequencing of the collected bioaerosols, we identified the species composition and relative abundance expression at different taxonomic levels during the two time periods. Besides, at the phylum classification level, the species with higher abundance expression in the morning sampling period included Proteobacteria, Bacteroidetes, and Firmicutes, while the species with higher abundance expression in the afternoon sampling period included Proteobacteria, Bacteroidetes, Firmicutes, Cyanobacteria, and Fusobacteria (Figure 2B). Meanwhile, the results of the species composition at the genus classification level demonstrated that Oscillospira, Clostridium, Lactobacillus, Shigella, Stenotrophomonas, Enterococcus, Alistipes, Escherichia, Trabulsiella, Christensenellaceae were the absolute dominant group in the morning sampling period, while Weissella, rhodoplanes, Staphylococcus, Sphingomonas, Paracoccus, Anaerotruncus, Zea, Lautropia, Fusobacterium, and Klebsiella were the absolute dominant group in the afternoon sampling period (Figure 2C). By analyzing the Alpha diversity indices of bacterial communities in bioaerosols during two distinct periods, it was observed that bioaerosols in the afternoon exhibited higher values for the Shannon index, observed OTUs, and PD whole tree (Figure 2D–G). These findings indicate a greater diversity of bacterial community in bioaerosols during the afternoon period. We further performed KEGG (Kyoto Encyclopedia of Genes and Genomes) pathway enrichment analysis on the bacterial genome data obtained from the sequencing samples. As shown in Figure S1 (Supporting Information), bioaerosols from garbage stations during the daytime period were involved in various cellular processes, metabolism, DNA replication and repair, carbohydrate metabolism, energy metabolism, signal transduction, and genetic information processing. These results proved that bioaerosols contained different genes related to certain infectious diseases or tumors, indicating the potential pathogenicity of the bioaerosols from garbage stations.

### 2.3 Bioaerosols from Garbage Stations Accelerated Vascular Aging in Mice

**Figure 3:**
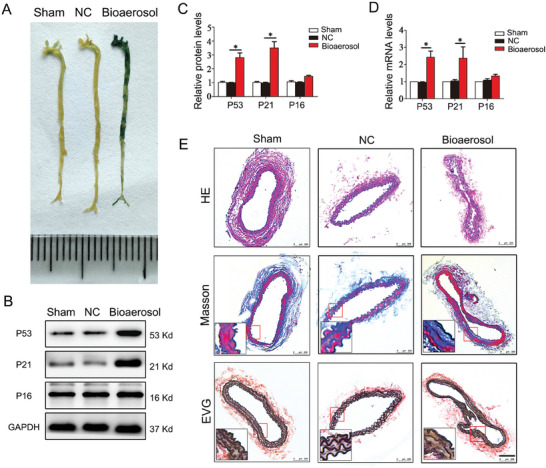
Effect of bioaerosols from garbage stations on vascular aging in C57BL/6 mice. A) Representative images of SA‐β‐Gal staining in cephalic artery. B) Representative Western blot of P53, P21, and P16. C) Quantitative analysis of P53, P21, and P16 protein. D) qPCR analysis of P53, P21, and P16 genes. E) The HE, Masson, and EVG staining of cephalic artery. *p < 0.05.

Senescence‐associated beta‐galactosidase (SA‐β‐Gal) staining revealed significant blue staining in mice subjected to nasal drip of bioaerosols compared to control groups, indicating the occurrence of vascular aging (**Figure** **3**A). Meanwhile, western blot and q‐PCR analyzed the expression of senescence‐related genes P53, P21, and P16 compared to the control mice. Western blot and q‐PCR analysis of P53 and P21 both revealed a marked increase in its expression in the cephalic artery of bioaerosols‐exposed mice, while the expression level of P16 was not statistically significant in the cephalic artery of bioaerosols‐exposed mice despite there was an up‐regulation trend (Figure 3B–D). Besides, HE, Masson, and EVG staining showed the lumen of the carotid artery was significantly enlarged, the collagen fiber content increased dramatically, and the elastic fibers had lost their normal concave and convex shape, suggesting that the cephalic artery was less flexible (Figure 3E). These results suggested that bioaerosols from garbage stations could accelerate vascular aging in C57BL/6 mice.

### 2.4 Bioaerosols from Garbage Stations Induced HUVECs Senescence and Impaired Endothelial Cell Function

**Figure 4:**
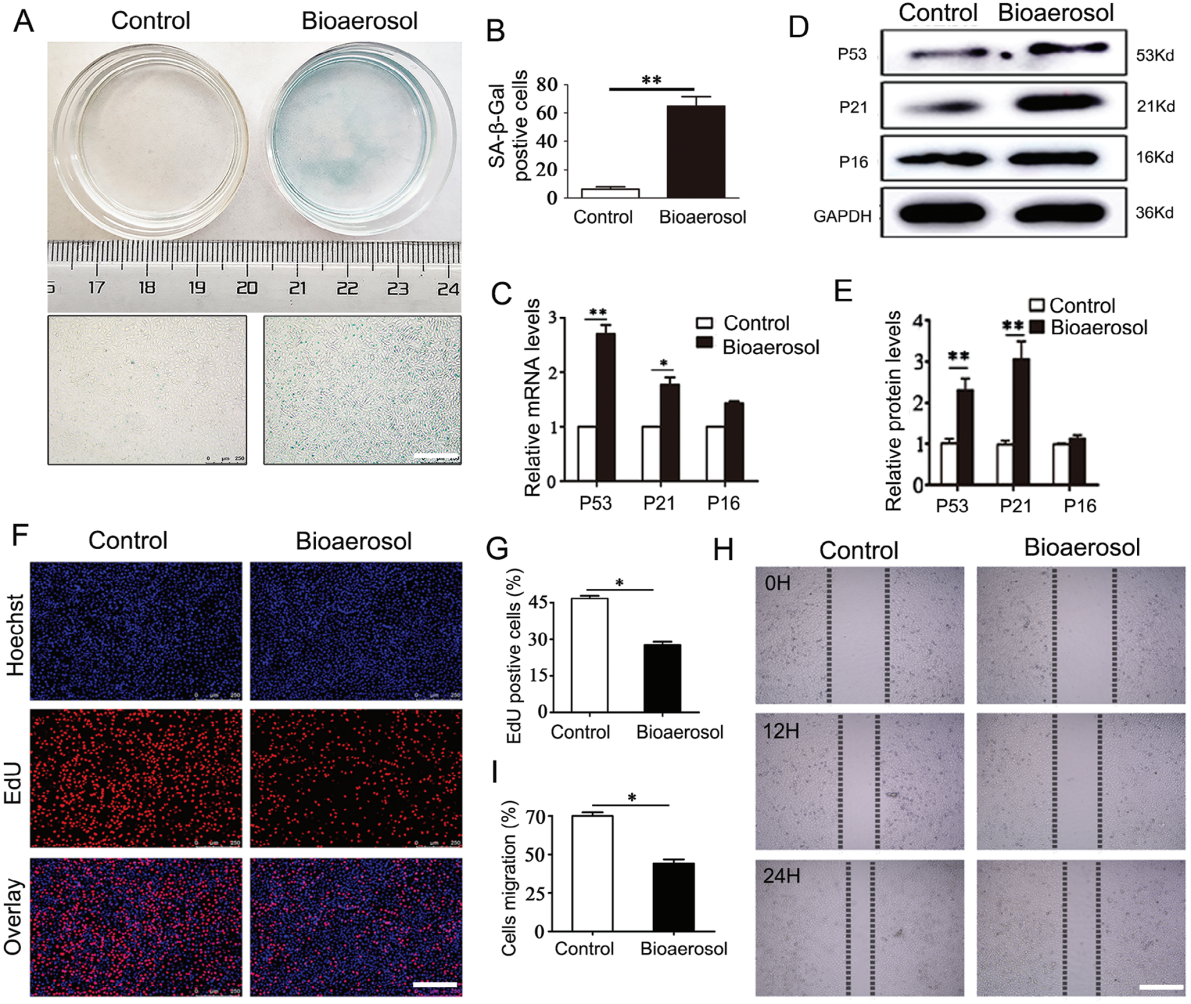
Effect of bioaerosols from garbage stations on the senescence of HUVECs. A,B) Representative images of SA‐β‐Gal staining and quantitative analysis. C) q‐PCR analysis of P53, P21, and P16 genes. D,E) Representative Western blot of P53, P21, and P16 and quantitative analysis. F,G) Representative images of EdU assay and quantitative analysis. H,I) Representative images of scratch assay and quantitative analysis. *p < 0.05, *p < 0.01.

Further in vitro studies were needed to explore the pro‐senescence effects of bioaerosols from garbage stations on vascular endothelial cell. Initially, we determined the concentration of bioaerosols at MOI = 10 using CCK‐8 assay as the experimental intervention condition (Figure S2, Supporting Information). Subsequently, we exposed HUVECs to bioaerosols from garbage stations for 48 hours and observed their effect on the senescence of HUVECs. The bioaerosols‐treated group had a higher number of visibly senescent cells and a significant increase in the percentage of stained positive cells compared with the control group (**Figure** **4**A,B). Furthermore, the intervention of bioaerosols resulted in a significant increase in the proportion of the G1 phase in the cell cycle of HUVECs, while the proportion of the S phase decreased significantly. These findings suggest that bioaerosols intervention had a G1 phase cell‐blocking effect (Figure S3, Supporting Information). To confirm the pro‐senescence effect of bioaerosols on HUVECs, we analyzed the mRNA and protein expression of senescence‐related genes (P53, P21, and P16) using q‐PCR and Western Blot, respectively. The results of our study showed that the expression levels of P16 mRNA and protein were not significantly different in the bioaerosols group (Figure 4C–E). However, the expression levels of P53 and P21 mRNA and protein were relatively greater and statistically significant. Endothelial cell senescence is characterized by reduced cell proliferation and migratory capabilities. We further investigated the changes in cell proliferation and migration capabilities of HUVECs after bioaerosols intervention. The EdU staining results revealed the reduction of proliferating HUVECs in the bioaerosols group (Figure 4F,G). Additionally, scratch migration experiments indicated that bioaerosols treatment significantly attenuated the migration ability of HUVECs (Figure 4H,I).

The above results of the experiment indicated that the intervention of bioaerosols impaired the proliferation and migratory capabilities of HUVECs. This is consistent with the characteristics of endothelial dysfunction and further supports the idea that bioaerosols promoted the senescence of HUVECs.

### 2.5 AS22a in Bioaerosols from Garbage Stations Promoted the Senescence of HUVECs

**Figure 5:**
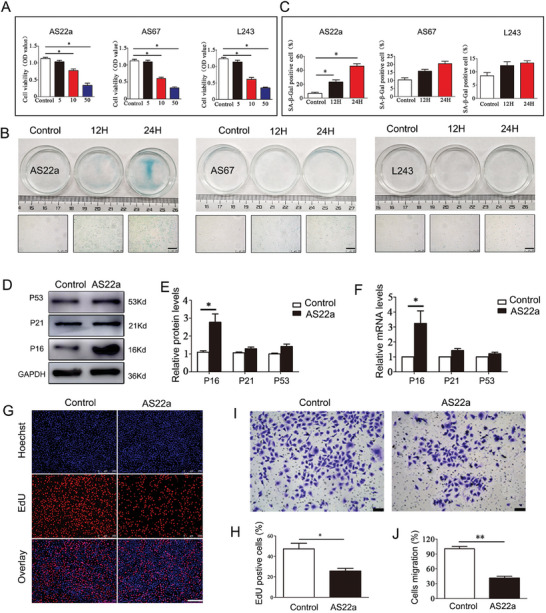
Effects of AS22a in bioaerosols from garbage stations on the senescence of HUVECs. A) Cell viability of HUVECs in different treatment groups. B,C) Representative image of SA‐β‐Gal staining and quantitative analysis. D,E) Representative Western blot of P53, P21, and P16 and quantitative analysis. F) qPCR analysis of P53, P21, and P16 genes. G,H) EdU staining and quantitative analysis. I,J) Transwell assay of HUVECs in different treatments and quantitative analysis. *p < 0.05, **p < 0.01.

Bacteria were collected using the sixth‐stage Anderson Aerosol Sampler, followed by isolation and purification procedures. Three bacteria exhibiting the highest relative abundance in the community were sequenced using 16S rDNA analysis. The results identified these bacteria as belonging to the genera Staphylococcus, Corynebacterium, and Desemzia. Subsequently, the isolated and purified strains were named AS22a, AS67, and L243 and uploaded to the NCBI database (Figure S4, Supporting Information). Next, we investigated the impact of three strains of bacteria on the senescence of HUVECs. To begin, we assessed the viability of HUVECs using the CCK‐8 assay and observed that at MOI = 10 or MOI = 50, the viability of HUVECs decreased significantly (**Figure** **5**A). This indicated that the three strains of bacteria had a clear toxic effect on HUVECs at these concentrations. For the subsequent experiment, we chose to pretreat HUVECs with an MOI of 5 for 24 h. Then, three strains of bacteria were utilized to intervene in HUVECs, and the results illustrated that the proportion of SA‐β‐Gal positive cells in HUVECs was highest after AS22a intervention, with a large number of blue‐stained senescent cells visible (Figure 5B,C). In contrast, AS67 and L234 interventions did not lead to significant changes. Based on these findings, AS22a (Staphylococcus caprae) was selected as the subject for subsequent research.

Additionally, flow cytometry was used to assess cell‐cycle alterations in HUVECs following AS22a intervention. The proportion of HUVECs in G1 phase increased significantly, while the proportion of S and G2 phases decreased remarkably. These findings suggested that AS22a intervention caused cell arrest in the G1 phase (Figure S5, Supporting Information). To assess the pro‐senescence impact of AS22a, we performed Western Blot and q‐PCR assays to measure mRNA and protein levels of age‐related genes (P53, P21, and P16). Our results indicated that mRNA and protein levels of P53 and P21 in the AS22a group were not significantly different from controls (Figure 5D–F). However, the age‐related gene P16 was significantly increased. Furthermore, we evaluated the effects of AS22a intervention on the proliferation and migration of HUVECs. For instance, the results of EdU staining indicated that the proportion of proliferating HUVECs in the AS22a intervention group decreased significantly compared to the control group (Figure 5G,H). Moreover, the transwell assays revealed that the cell migration of the AS22a treatment group decreased considerably (41.65% ± 0.07) in comparison with the control group (100.59% ± 0.13) (Figure 5I,J). These findings suggested that the presence of AS22a in bioaerosols from garbage stations promoted the senescence of HUVECs.

### 2.6 AS22a Strain in Bioaerosols from Garbage Stations Accelerated Vascular Aging in Mice

**Figure 6:**
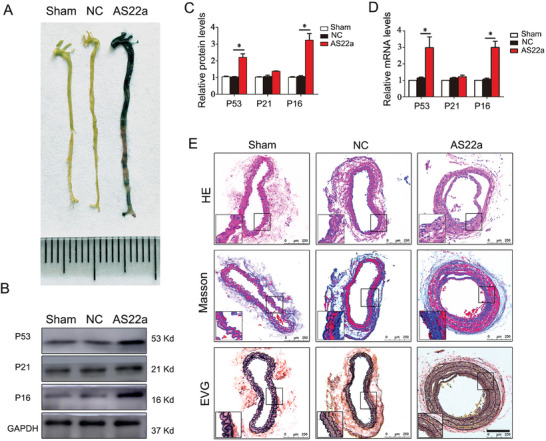
Effect of AS22a strain in bioaerosols from garbage stations on vascular aging in C57BL/6 mice. A) Representative images of SA‐β‐Gal staining in the cephalic artery. B,C) Representative Western blot of P53, P21, and P16 and quantitative analysis. D) qPCR analysis of P53, P21, and P16 genes. E) The HE, Masson, and EVG staining of cephalic artery. *p < 0.05.

The AS22a nasal drip group exhibited notably increased blue staining in vessels compared to the NC group, indicating the occurrence of vascular aging (**Figure** **6**A). Further analysis of senescence‐related genes, including P53, P21, and P16, was performed via Western Blot and q‐PCR experiments. There was a significant up‐regulation of the mRNA and protein expression levels of P53 and P16 in the AS22a intervention group as compared to the NC group, which was found to be statistically significant (Figure 6B–D). However, the senescence gene P21 did not show a statistically significant difference, although there was a noticeable trend toward an increase. Finally, we observed changes in the intima, collagen fibers, and vascular elastic fibers at the cephalic artery of mice using HE, Masson, and EVG staining. The staining revealed that the intima was thicker, the collagen fiber content had significantly increased, and the elastic fibers had become flat and lost their normal concave shape when compared to the NC group (Figure 6E). As described, chronic exposure to AS22a nasal drip in mice accelerated histological changes in their arteries, leading to increased deposition of collagen fibers, deformed elasticity of fibers, and thickening of arterial walls. These findings confirm that AS22a exposure accelerates vascular aging.

### 2.7 AS22a Induced Vascular Endothelial Senescence by Activating the NF‐κB/NLRP3 Signaling Pathway

**Figure 7:**
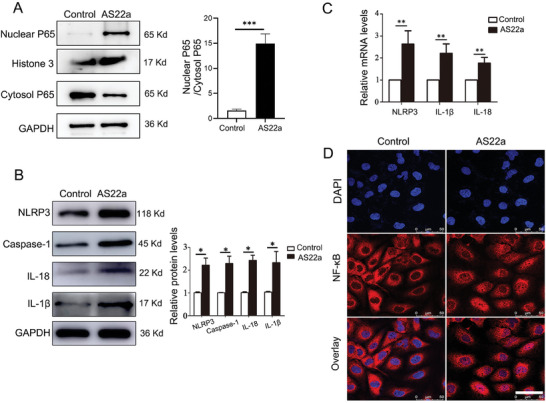
AS22a activated NF‐κB and NLRP3 inflammasomes. A) Representative western blot of NF‐κB (p65) in both the cytoplasm and nucleus, and quantitative analysis. B) Representative western blot of NLRP3, Caspase‐1, IL‐1β, and IL‐18 and quantitative analysis. C) q‐PCR analysis of NLRP3, IL‐1β, and IL‐18 genes. D) Representative immunofluorescence images of staining of NF‐κB. *p < 0.05, **p < 0.01, ***p < 0.001.

Functional predictions of the AS22a genome in host cells were made using 16S rRNA sequencing analysis. As shown in Figure S6 (Supporting Information), the KEGG second‐level classification revealed that genes of AS22a are involved in various processes, including carbohydrate metabolism, amino acid metabolism, energy metabolism, and replication and repair in genetic information processing. Additionally, the genome contained genes associated with aging and cardiovascular diseases. These functional predictions further support the notion that AS22a promotes vascular aging. After establishing that chronic exposure to AS22a can accelerate vascular aging, we delved into the potential molecular mechanisms involved. The report suggests that microorganisms may facilitate the assembly and activation of NLRP3 inflammasomes by increasing the transcriptional activity of NF‐κB, thereby inducing inflammatory responses. We aimed to determine if AS22a treatment of vascular endothelial cells activates NK‐κB signaling and NLRP3 inflammasomes, as inflammation is a crucial pathological mechanism of senescence. Subsequently, we conducted a western blot assay to analyze the protein expression of NK‐κB (p65) in both the cytoplasm and nucleus, and the result showed that AS22a treatment markedly increased p65 nuclear translocation relative to the control group (**Figure** **7**A). After intervening with AS22a, we observed an increase in mRNA and protein expression in downstream‐related genes of NLRP3, including Caspase‐1, IL‐1β, and IL‐18 (Figure 7B,C). To further confirm the activation effect of AS22a on NF‐κB, we conducted an immunofluorescence analysis to observe the intracellular distribution of the NF‐κB subunit p65. The p65 subunit of NF‐κB was predominantly localized to the cytoplasm of HUVECs in the control group. However, in the AS22a treatment group, it was primarily found in the nucleus of HUVECs. These results suggested that AS22a intervention promoted the transfer of NF‐κB subunit p65 to the nucleus of endothelial cells, which was a marker for activating NF‐κB (Figure 7D). The findings indicated that AS22a has the ability to activate NF‐κB and NLRP3 inflammasomes in HUVECs, consequently stimulating the synthesis and release of inflammatory factors such as IL‐1β and IL‐18.

**Figure 8:**
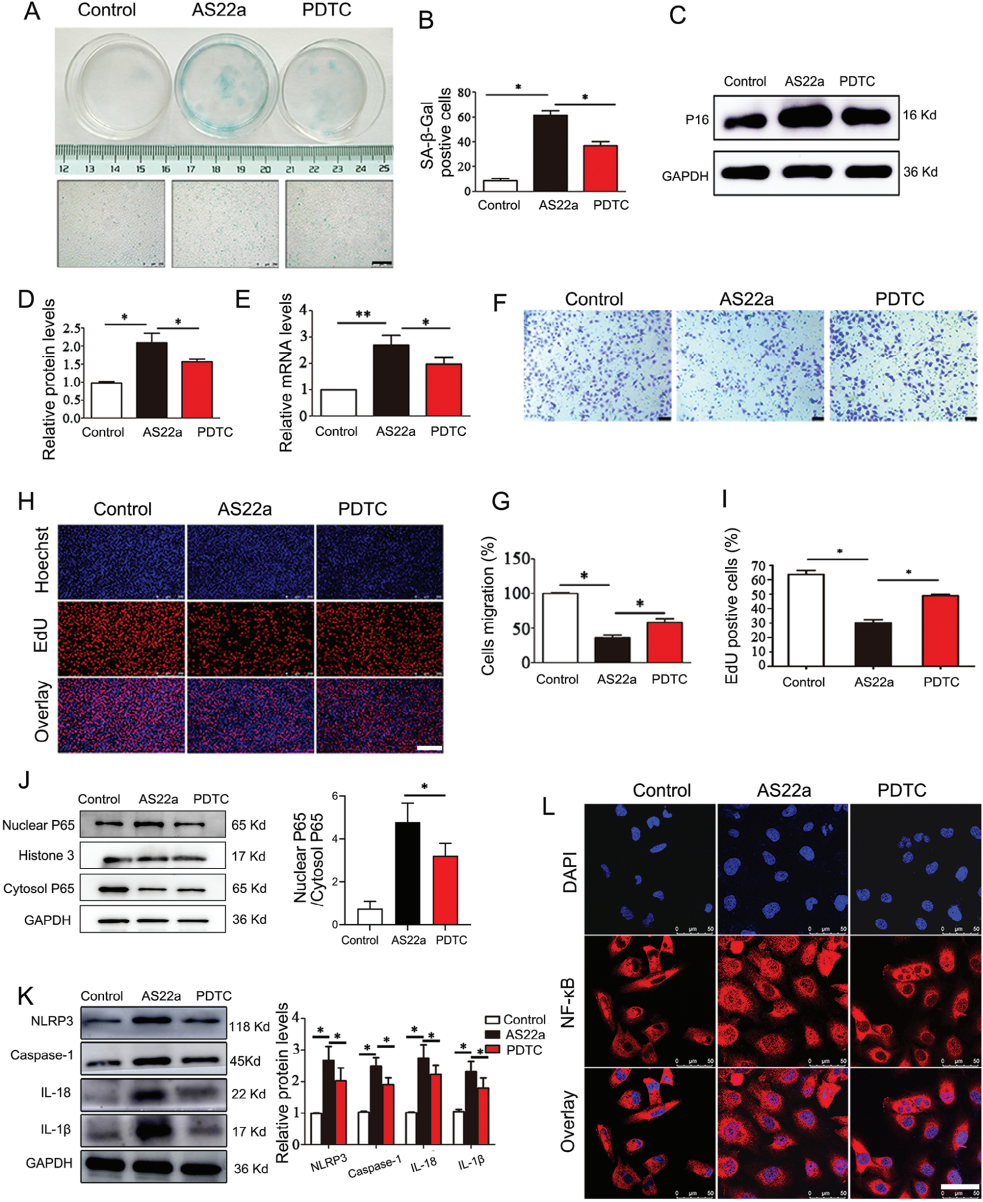
NF‐κB/NLRP3 signaling was involved in AS22a‐stimulated senescence of HUVECs. B) Representative images of SA‐β‐Gal staining in HUVECs and quantitative analysis. C,D) Representative western blot analysis of P16 protein and quantitative analysis. E) q‐PCR analysis of the P16 gene. F,G) Transwell assay for different treatments and quantitative analysis. H,I) EdU assay and quantitative analysis. J) Representative western blot of NF‐κB (p65) in both the cytoplasm and nucleus, and quantitative analysis. K) Representative Western blot analysis of NLRP3, Caspase‐1, IL‐1β, and IL‐18 and quantitative analysis. L) Representative immunofluorescence images of staining of NF‐κB. *p < 0.05, **p < 0.01.

Based on the above findings, PDTC (NF‐κB inhibitor) and AS22a acted together in HUVECs to further clarify whether NF‐κB/NLRP3 signaling mediated the senescence of HUVECs stimulated by AS22a. SA‐β‐Gal staining showed that the proportion of blue staining in HUVECs decreased statistically significantly after PDTC treatment (**Figure** **8**A,B). Typically, we analyzed the levels of P16 protein and mRNA in HUVECs and observed a decrease in cells treated with PDTC compared to those treated with AS22a (Figure 8C–E). We also conducted Transwell and EdU proliferation experiments, which revealed different restoration of proliferation and migration in HUVECs after PDTC treatment, indicating functional changes induced by the intervention (Figure 8–I). Next, we analyzed the protein expression of NK‐κB (p65) in both the cytoplasm and nucleus after PDTC and AS22a coculture with HUVECs. We observed a significant reduction in p65 nuclear translocation following PDTC treatment, in comparison to the AS22a treatment group (Figure 8J). Furthermore, we measured the expression levels of NLRP3 and downstream inflammatory factor proteins using Western Blot. Our findings indicated that the protein levels of NLRP3, Caspase1, IL‐1β, and IL‐18 in HUVECs were significantly decreased after the addition of PDTC (Figure 8K). Moreover, immunofluorescence analysis revealed changes in the intracellular distribution of NF‐κB subunit p65 after the addition of PDTC. Hence, PDTC can attenuate the nuclear transition of NF‐κB subunit p65 to cells when compared to the AS22a treatment group (Figure 8L). Together, these observations suggested that the NF‐κB/NLRP3 signaling pathway was involved in AS22a‐induced HUVEC senescence through the regulation of inflammatory factors.

## 3 Discussion and Conclusions

In this study, we found that there were high concentrations of conditioned pathogenic bioaerosols within the household garbage station environment, and chronic high‐concentration exposure could accelerate vascular aging in mice. To further investigate the pathogenic causes, we isolated and identified a bacterial strain (Staphylococcus ligation AS22a) in the bioaerosols, and found that it can regulate the inflammatory response through NF‐κB/NLRP3 signaling to stimulate the occurrence of vascular aging. To this end, we first demonstrated that the bioaerosol component of PM2.5 can accelerate vascular aging via the NF‐κB/NLRP3 signaling pathway.

Prior studies have established a strong correlation between the concentration and diameter of airborne particulate matter and the occurrence of acute myocardial infarction and stroke.^[^ ^15^ ^]^ A prospective cohort study showed a causal association between long‐term PM2.5 exposure and the occurrence of ischemic heart disease, heart failure, hypertension, arrhythmia, and other adverse events.^[^ ^16^ ^]^ PM2.5 exposure resulted in increased oxidative stress in the lungs and heart, increased inflammation in the lungs and throughout the body,^[^ ^17^ ^]^ decreased oxygen saturation, and decreased vascular endothelial repair capacity.^[^ ^18^ ^]^ Our analysis of 8144 participants with PM2.5 exposure confirmed an elevated risk of Major Adverse Cardiovascular Events (MACE) and cardiovascular disease (CVD), consistent with previous findings. Bioaerosols, as an important environmental exposure in PM2.5, have attracted more and more attention in recent years. Epidemiological studies have demonstrated that chronic exposure to bioaerosol can increase the incidence of respiratory, circulatory, and digestive diseases.^[^ ^19^ ^]^ In our study, the garbage station selected primarily serves as a temporary storage facility for household waste generated by nearby residents, predominantly comprising kitchen waste, and is built indoors with only one entrance and exit channel, with average ventilation conditions. These factors collectively create favorable conditions for microbial proliferation, thereby partially explaining the high concentration of bioaerosols present in this environment.

At the same time, the analysis of the samples indicated that a majority of the bioaerosol particles found in garbage stations were small enough to be inhaled into the lower respiratory tract and deposited in the alveoli. Moreover, some of these particles had the potential to enter the bloodstream through the gas exchange, leading to multi‐system dissemination.^[^ ^20^ ^]^ Sequencing analysis also showed that the bioaerosols in the garbage station contained a high concentration of opportunistic bacteria, which was confirmed in the follow‐up experiment. In summary, the environment of the household garbage station at the sampling site is filled with high concentrations of bioaerosols, containing various conditional pathogenic bacteria. The small aerodynamic diameters of these bioaerosols enable them to be inhaled and deposited in the lungs. Chronic exposure to such bioaerosols may carry potential toxic effects on various organ systems in the human body, presenting a significant health threat.

Multiple studies have confirmed that repeated exposure to bioaerosols can induce chronic inflammatory responses in the body, increasing the risk of cardiovascular events.^[^ ^19^, ^21^ ^]^ Moreover, there is a close relationship between inflammation and vascular aging, with chronic inflammation being regarded as a significant contributor to pathological vascular aging.^[^ ^22^ ^]^ The factors secreted by senescent cells can promote chronic inflammation and induce cellular senescence of normal cells. Meanwhile, chronic inflammation can accelerate the senescence of immune cells, leading to weakened immune function and the inability to clear senescent cells and inflammatory factors, forming a vicious cycle of inflammation and cellular senescence inside the body.^[^ ^23^ ^]^ Hamanaka and Mutlu suggested that prolonged exposure to organic pollutants can induce chronic inflammation in the body, accelerating the onset of age‐related degenerative diseases.^[^ ^24^ ^]^ Yue et al. indicated that exposure to air pollution increases the concentration of intercellular adhesion molecule‐1 and vascular cell adhesion molecule‐1, promotes the expression of inflammatory genes, and leads to abnormal methylation.^[^ ^25^ ^]^ Pope et al. found that short‐term PM2.5 exposure promoted apoptosis of vascular endothelial cells and elevated levels of inflammatory cells.^[^ ^7a^ ^]^ The focus of our study lies in the examination of bioaerosols found in PM2.5 and their impact on vascular aging through a specific underlying mechanism associated with chronic inflammation in the body.

The NLRP3 inflammasome acts as a receptor within cellular innate immune systems, capable of recognizing and responding to diverse danger signals.^[^ ^26^ ^]^ The activated NLRP3 inflammasome can induce the expression and release of proinflammatory factors such as IL‐1β and IL‐18, playing a pivotal role in the pathogenesis of atherosclerosis, hypertension, diabetes, and other inflammation‐related diseases.^[^ ^27^ ^]^ In the D‐galactose‐induced endothelial cell senescence model, there was an increase in the expression of NLRP3 inflammasome, IL‐1β and IL‐18. However, inhibition of NLPR3 inflammasome can alleviate endothelial cell aging and improve endothelial cell function to a certain extent.^[^ ^12a^ ^]^ A large number of studies have confirmed that microorganisms are one of the most common exogenous factors that stimulate the activation of NLRP3 inflammasome in the organism. When microorganisms invade the organism, they are promptly recognized by the NLRP3 inflammasome.^[^ ^28^ ^]^ This recognition leads to the synthesis and release of inflammatory factors, recruitment of inflammatory cells, and participation in the organism's inflammatory response.^[^ ^12b^ ^]^ These processes are crucial for maintaining the organism's defense function, indicating that the NLRP3 inflammasome plays a significant role in aging. In this study, we isolated and purified one strain exhibiting the highest relative abundance in the bioaerosol using 16S rDNA analysis and also demonstrated that AS22a stimulation increases the expression of NLRP3, caspase‐1, IL‐1β, and IL‐18 during the senescent process of HUVECs, consistent with previous findings.

NF‐κB is a nuclear transcription factor, representing another crucial signal in the inflammatory response. The levels and activity of NF‐κB are elevated in aging cells and tissues, and the inhibition of its activity has shown potential for improving or alleviating the aging process.^[^ ^29^ ^]^ Additionally, the overactivation of NF‐κB leads to the upregulation of cytokines such as interleukin IL‐1β, adhesion molecules, immune receptors, and inflammation‐related enzymes. This ultimately exacerbates the inflammatory response.^[^ ^30^ ^]^ Some previous studies have shown that microorganisms can mediate the assembly and activation of NLRP3 inflammatory by upregulating the transcriptional activity of NF‐κB, thereby promoting the inflammatory response.^[^ ^31^ ^]^ These findings suggest that NF‐κB and NLRP3 are closely related to the activation of inflammation. Based on the KEGG second‐level classification results and the presence of genes associated with aging and cardiovascular diseases in the genome, this study aimed to investigate whether AS22a stimulation activates the NF‐κB signaling pathway in endothelial cell senescence. As expected, activation of the NF‐κB signaling pathway was observed during AS22a‐induced senescence of HUVECs. Furthermore, upon further treatment with the NF‐κB inhibitor PDTC, the senescent effects of AS22a on HUVECs were attenuated, accompanied by downregulation of NLRP3, IL‐1β, and IL‐18. The above findings collectively indicated that the NF‐κB signaling pathway regulates the secretion of pro‐inflammatory cytokines IL‐1β and IL‐18 via the NLRP3 inflammasome, thereby facilitating the mediation of AS22a‐induced pro‐aging effects on HUVECs.

The garbage stations established in urban areas primarily serve as temporary storage for various types of waste generated by the surrounding population in their daily lives.^[^ ^32^ ^]^ Generally, the waste in urban garbage stations consists mainly of a large amount of kitchen waste, which provides an excellent environment for microbial growth. Consequently, this leads to a noticeable increase in microbial concentration in the air around the garbage stations. However, the impact of the bioaerosols emitted by these stations on human health has long been neglected. Our findings indicated that the presence of high concentrations of conditionally pathogenic bioaerosols in PM2.5 from garbage stations, and chronic bioaerosols exposure could accelerate vascular aging in mice. AS22a, as a bacterial component of bioaerosols from garbage stations, could stimulate vascular endothelial senescence via the NF‐κB/NLRP3 signaling pathway. This study first proved that bioaerosols in PM2.5 could accelerate vascular endothelial senescence via the NF‐κB/NLRP3 signaling pathway. This not only suggests that chronic exposure to high concentrations of harmful bioaerosols in PM2.5 be harmful to human health which could be manifested as a toxic effect of accelerating vascular endothelial senescence, but also provides a reference for the risk assessment of occupational exposure in sanitation industries. Governments could significantly improve public health by implementing appropriate interventions to reduce the risk of cardiovascular disease from air pollution near household garbage stations.

## 4 Experimental Section

### Clinical Data of the PM2.5 on Cardiovascular Outcomes Incidence

This prospective cohort study was based on survey data from the China Health and Retirement Longitudinal Study (CHARLS) with ethics approval from the Ethical Review Committee of Peking University (No. IRB00001052‐11015).^[^ ^33^ ^]^ From the CHARLS cohort study, participants were included who had complete demographic information and blood tests. We excluded participants who had previous cardiovascular disease (CVD) or stroke (*n* = 1463), did not have follow‐up event data (*n* = 307), less than 45 years old (*n* = 193), and did not have information for covariates (*n* = 24). The final sample size for the analysis of PM2.5 exposure and cardiovascular outcomes was 8144 participants.

The CVD or stroke events were assessed by individuals who self‐reported “yes” to the question “Have you been diagnosed with heart attack, coronary heart disease, angina, congestive heart failure, or other heart problems by a doctor?” or “Have you been diagnosed with stroke by a doctor?.”^[^ ^34^ ^]^ Major adverse cardiovascular event (MACE) incidence refers to the first ever CVD or stroke event. The average annual PM2.5 exposure was calculated using daily full‐coverage PM2.5 data at a spatial resolution of 10 km, which is publicly available from Tracking Air Pollution in China (TAP, http://tapdata.org.cn/).^[^ ^35^ ^]^ Annual mean PM2.5 concentrations across study sites ranged from 17.14 to 112.65 µg m^−3^, with 3 quartile cutoff points of 46.92, 65.73, and 86.99 µg m^−3^. Multivariate logistic regression analysis was performed for MACE incidence adjusting for age, gender, education, residence, body mass index, smoking status, alcohol consumption, hypertension status, diabetes status, dyslipidemia status, kidney disease status at baseline. A restricted cubic spline model with 4 knots was performed to estimate the dose‐response functions of PM2.5 exposures and MACE incidence.

### Collection of Bioaerosols from Garbage Stations

Bioaerosol samples were taken from the air at a household trash transfer station in Guangzhou, China (23.18° N, 113.33° E), and airborne bioaerosols were collected using an Andersen six‐stage microbial activity impingement sampler (Junray, China). The brief steps were as follows: First, thoroughly cleanse the sampler with neutral detergent and deionized water, repeating the process three times. Concurrently, immerse the Andersen six‐stage microbial activity impingement sampler in 75% alcohol for disinfection. Next, position the sampling equipment approximately 2 m away from the central area of the waste station, with a height of about 1.5 m from the ground. Set the air sampling flow rate to 28.3 L m^−3^, with each sample collected for a duration of 30 min. Repeat the sampling process five times to ensure a more accurate characterization of bacterial aerosols in the air environment. Following sampling, the Petri dishes were taken out and transported to a 37 °C incubator (Shanghai Yiheng, China), where they were incubated for 24–48 h before the bacterial colony counts were taken to conduct subsequent experiments.

### Sample DNA Extraction and Detection

We chose to use phenol‐chlorine extraction to extract the DNA from the samples. Initially, the sample suspension was incubated at 37 °C for 30 min with lysozyme (Servicebio, China) and then subjected to centrifugation with lysate. Subsequently, the sample DNA was analyzed using SimpliNano analyzers (GE, USA), and its purity was assessed by spectrophotometric measurement of the A260/A280 ratio. Finally, the integrity of the sample DNA was verified by 1% agarose gel electrophoresis.

### Illumina Sequencing

The DNA gel strips were collected, and the concentrations were adjusted to homogenize across groups after relative quantification to reduce sequencing errors. The DNA samples were then purified using the Illuminate Paired‐end Library Preparation Kit (Illumina, China), and the end products were sequenced on the Illumina HiSeq2000 platform (Ribobio, China).

### Bioinformatics Analysis

The representative sequences of each sample's operational taxonomic units (OUTs) were verified and calibrated using the PyNAST algorithm to further generate phylogenetic evolutionary trees, followed by α‐diversity analysis. Based on the inferred species composition, a comparative analysis of the relative abundances across various taxonomic levels was conducted. Subsequently, heat maps were generated to illustrate the differences between samples through color gradient changes.

### Chronic Exposure of C57BL/6 Mice with Bioaerosols from Garbage Station

C57BL/6 mice (male, 20 weeks) were exposed to the bioaerosols suspension via the conventional nasal drip route at a concentration of 1 × 10^5^ CFU mL^−1^ and 30 µL per mouse. After 6 months, the cephalic artery and aorta of the mice were isolated, and the vessels were cleaned and preserved in 4% paraformaldehyde solution for 24 h.

### Histopathological Analysis

The fixed vascular specimens underwent a dewaxing gradient procedure, xylene transparent treatment, overnight immersion in wax solution, and subsequently embedded by an embedding machine (Sakura, Japan). Slices of 4 mm thickness were serially sectioned from the embedded wax blocks. Subsequently, the tissue sections were subjected to staining with HE, Masson, and EVG stains, and microscopic images were captured using a microscope (Leica, Germany) to assess alterations in collagen and elastic fibers.

### Senescence‐Associated β‐Galactosidase (SA‐β‐gal) Staining

Initially, the cells and tissues were fixed with 4% paraformaldehyde, and then they were detected with pre‐prepared SA‐β‐Gal staining solution (Beyotime, China) according to the manufacturer's instructions and incubated at 37 °C for 18 h. Afterward, the staining solution was discarded, and the samples were washed three times with PBS buffer. Subsequently, fluorescence microscopy (Leica, Germany) was employed to observe the cells and tissues. Finally, the ratio of positive cells to the total cells within the field of view was calculated.

### Flow Cytometry Analysis of the Cell Cycle

The amount of DNA in the cells was measured to determine the period of mitosis as well as being able to reflect the proliferation of the cells. In detail, the cells after treatment were digested and collected, followed by centrifugation at 1000 rpm for 5 min to obtain the cell precipitate. Then, 500 µL of PI staining solution was added to each sample and incubated for 30 min at room temperature. After incubation, cells were collected for flow cytometry analysis (Beckman, USA) of fluorescence intensity, and the data were recorded and analyzed.

### Cell Viability Assay

The cells were seeded in 96‐well plates. After the cell pretreatment was done, the CCK‐8 working solution (Beyotime, China) was added to each well. Then the 96‐well plates were transferred to a cell incubator to continue incubation for 2 hours. Finally, cell viability was measured by a microplate reader (Thermo Fisher, USA) at the absorbance of 450 nm.

### EdU Detection

The cells were fixed using 4% paraformaldehyde for 30 min at room temperature, and the proliferating cells were detected using a one‐step EdU staining kit (RiboBio, China) following the manufacturer's instructions. Subsequently, the cells were counterstained with Hoechst (RiboBio, China), and the EdU‐positive cells were visualized using a fluorescence microscope (Leica, Germany).

### Cell Scratch Assay

The cells were seeded into six‐well plates. Once the cells reached over 85% confluence, the monolayer of HUVECs was scraped using a 200µL micropipette tip. The scratch images were taken under the light microscope (Leica, Germany) immediately after the scratching was completed. Then the six‐well plate was placed back into the cell incubator and incubated for 24 h. After that, the scratch images were again taken under the microscope at the previously photographed positions after 24 hours of incubation. The scratch migration rate was calculated as follows: Migration rate (%) = (*A*0 ‐ *A*n)/A0 ×100%. *A*0 represents the initial wound area, and an represents the final wound area.

### Transwell Assay

The cells were plated at the density of 10 × 10^4^ cells per well in the top chamber of a transwell insert and cultured in the medium for 12 h. After that, fixed the cells with 4% paraformaldehyde for 20 min and then stained with 1% aqueous‐methanol solution for 30 min at 37 °C. Finally, the cell staining was visualized using a Leica microscope (Leica, Germany), and the number was counted by ImageJ software.

### Immunofluorescence

The cells were fixed with 4% paraformaldehyde for 30 min, followed by permeabilized by 0.1% Triton X‐100 (Sigma‐Aldrich, USA) for 10 min and then blocked the nonspecific binding by 10% fetal bovine serum (Solarbio, China) for 30 min. Subsequently, the cells were incubated with specific primary antibodies NF‐κB (1:400, Abcam, USA) overnight at 4 °C and following incubated with a goat anti‐rabbit Dylight 488‐conjugated secondary antibody (1:200, Abcam, USA) at 37 °C for 2 h. After washing with PBS, the cells with DAPI for 30 min. Finally, the images were taken by a confocal laser microscope (Leica, Germany).

### qRT‐PCR Analysis

Total RNA was extracted from HUVECs using TRIzol reagent (AG, China). The concentration of RNA was measured through a MicroplateReader (GE, USA). Following the manufacturer's instructions, cDNA was synthesized using a PrimeScript reagent kit (Takara, Japan). Subsequently, the cDNA samples were used for qRT‐PCR detection of target gene expression using (Takara, Japan). Data were analyzed with the Livak method to quantify relative to the aiming gene expression.

### Western Blot

The cells and tissues were lysed in RIPA lysis buffer with 0.1 × 10^−3^ m proteinase inhibitor (Beyotime, China). Protein concentration was determined using a BCA protein assay. Subsequently, 5% SDS‐PAGE was employed to separate the protein samples, followed by transferring protein to a polyvinylidene difluoride membrane (Millipore, USA). Afterward, the membranes were incubated in 5% skim milk (GBCBIO, China) for 1 hour at 37 °C, and subsequently incubated with specific primary antibodies including P53 (Abcam, USA), P21 (Abcam, USA), P16 (Abcam, USA), and GAPDH (Abcam, USA) overnight at 4 °C. On the following day, the membranes were incubated with corresponding secondary antibodies. The protein bands were detected by color fluorescence imaging technology (GE, USA) and analyzed using Image J software.

### Statistical Data Analysis

All results were presented as mean ± standard deviation (SD), and the normality of all data was assessed. A T‐test or one‐way ANOVA was conducted to compare the two groups. If the variances were the same, the LSD method was applied. Nevertheless, if the variance was not uniform, the data must be log‐transformed before analysis. SPSS software (version 22.0) was used for statistical analysis. All statistical tests were two‐sided and differences were statistically significant at *P* < 0.05.

## Conflict of Interest

The authors declare no conflict of interest.

## Supporting information



Supporting Information

## Data Availability

The data that support the findings of this study are available from the corresponding author upon reasonable request.
