# Supplementary material for: Chronic Exposure to Bioaerosols in PM2.5 from Garbage Stations Accelerates Vascular Aging via the NF‐κB/NLRP3 Pathway
Source: Adv Sci (Weinh). 2024 Oct 22;11(45):2404142. doi: 10.1002/advs.202404142 (PMC11615796; doi:10.1002/advs.202404142)
Supplement: Supplementary file 1 — Supporting Information [file ADVS-11-2404142-s001.docx]

Supporting Information

**Chronic Exposure to** **Bioaerosols in PM2.5 from Garbage Stations Accelerates Vascular Aging via the NF-κB/NLRP3 Pathway**

*Peier Chen, Xiaodong Ning, Weijing Feng, Yajing Li, Guoqin Chen, Xu Shi, YuXuan Pan, Xueqin Shi, Yafang Xiao, Yuhua Liu, Guoxia Zhang^∗^, Feiran Zhou^∗^, Caiwen Ou^∗^*

P. Chen, X. Ning, Y. Li, X. Shi, Y. Pan, X. Shi, Y. Xiao, C. Ou

The Tenth Affiliated Hospital (Dongguan People's Hospital), The First School of Clinical Medicine, Southern Medical University, Dongguan 523059, China

E-mail: oucaiwen@smu.edu.cn

W. Feng

Department of Cardiology, State Key Laboratory of Organ Failure Research, Guangdong Provincial Key Laboratory of Cardiac Function and Microcirculation, Nanfang Hospital

Southern Medical University, Guangzhou 510515, China

G. Chen

Department of Cardiology, The Affiliated Panyu Central Hospital of Guangzhou Medical University, Guangzhou 511400, China

Y. Liu

Department of General Practice, The Tenth Affiliated Hospital (Dongguan People's Hospital),

Southern Medical University, Dongguan 523059, China

G. Zhang

Department of Environmental Health, Guangdong Provincial Key Laboratory of Tropical Disease Research, School of Public Health, Southern Medical University, Guangzhou 510515, China

E-mail: guoxiazhang@smu.edu.cn

F. Zhou

Department of Cardiology, The First Hospital of Hunan University of Chinese Medicine, Changsha 410007, China

1. mail: 2023681085@gzhmu.edu.cn

| **Variables** | **Total**  **(n=8144)** | **Quartile 1**  **(n=2097)** | **Quartile 2 (n=2019)** | **Quartile 3 (n=2056)** | **Quartile 4**  **(n=1972)** | ***p Value*** |
| --- | --- | --- | --- | --- | --- | --- |
| **PM2.5,** **μg/m^3^, mean (SD)** | 65.9 (23.6) | 35.9 (8.78) | 55.8 (5.92) | 77.8 (5.67) | 95.8 (6.96) | 0.000 |
| **Age, yrs, mean (SD)** | 58.6 (9.3) | 58.0 (9.4) | 59.1 (9.4) | 58.8 (9.5) | 58.6 (8.8) | 0.001 |
| **Gender, n (%)** |  |  |  |  |  | 0.202 |
| **Male** | 3833 (47.1) | 1011 (48.2) | 926 (45.9) | 992 (48.2) | 904 (45.8) |  |
| **Female** | 4311 (52.9) | 1086 (51.8) | 1093 (54.1) | 1064 (51.8) | 1068 (54.2) |  |
| **BMI, kg/m^2^, mean (SD)** | 23.3 (3.39) | 22.8 (3.35) | 23.0 (3.31) | 23.5 (3.34) | 24.0 (3.43) | 0.000 |
| **Smoke, n (%)** | 3197 (39.3) | 833 (39.7) | 810 (40.1) | 796 (38.7) | 758 (38.4) | 0.653 |
| **Alcohol consumption last year, n (%)** | 2770 (34.0) | 684 (32.6) | 727 (36.0) | 704 (34.2) | 655 (33.2) | 0.111 |
| **SBP, mmHg, mean (SD)** | 129.0 (21.1) | 129.7 (21.8) | 127.7 (20.4) | 128.8 (21.8) | 129.8 (20.4) | 0.006 |
| **DBP, mmHg, mean (SD)** | 75.1 (12.1) | 75.8 (12.4) | 74.3 (11.9) | 74.5 (12.1) | 75.8 (11.9) | 0.000 |
| **Glucose, mg/dl, mean (SD)** | 109.8 (35.8) | 107.0 (35.0) | 108.4 (32.0) | 111.0 (36.8) | 112.8 (38.8) | 0.000 |
| **Creatinine, mg/dl, mean (SD)** | 0.78 (0.22) | 0.80 (0.19) | 0.80 (0.31) | 0.76 (0.20) | 0.74 (0.18) | 0.000 |
| **TG, mg/dl, mean (SD)** | 132.5 (110.5) | 136.9 (118.8) | 132.7 (117.5) | 131.9 (110.1) | 128.1 (92.8) | 0.551 |
| **LDL-C, mg/dl, mean (SD)** | 116.0 (35.0) | 117.6 (37.3) | 117.0 (35.2) | 113.2 (33.6) | 116.2 (33.4) | 0.001 |
| **Education, n (%)** |  |  |  |  |  | 0.000 |
| **Primary to middle school** | 7394 (90.8) | 1965 (93.7) | 1760 (87.2) | 1908 (92.8) | 1761 (89.3) |  |
| **High school or above** | 752 (9.2) | 132 (6.3) | 259 (12.8) | 148 (7.2) | 213 (10.7) |  |
| **Residence, n (%)** |  |  |  |  |  | 0.000 |
| **Urban** | 2709 (33.3) | 557 (26.6) | 786 (38.9) | 616 (30.0) | 750 (38.0) |  |
| **Rural** | 5435 (66.7) | 1540 (73.4) | 1233 (61.1) | 1440 (70.0) | 1222 (62.0) |  |
| **History of comorbidities, n (%)** |  |  |  |  |  |  |
| **Hypertension** | 1814 (22.3) | 422 (20.1) | 444 (22.0) | 484 (23.5) | 464 (23.5) | 0.024 |
| **Diabetes** | 414 (5.1) | 74 (3.5) | 103 (5.1) | 105 (5.1) | 132 (6.7) | 0.000 |
| **Dyslipidemia** | 715 (8.8) | 134 (6.4) | 166 (8.2) | 189 (9.2) | 226 (11.5) | 0.000 |
| **Kidney disease** | 355 (4.4) | 100 (4.8) | 85 (4.2) | 93(4.5) | 77 (3.9) | 0.560 |
| **History of medication use, n (%)** |  |  |  |  |  |  |
| **Hypertension medications** | 1308 (16.2) | 281 (13.5) | 317 (15.8) | 362 (17.7) | 348 (17.8) | 0.000 |
| **Diabetes medications** | 251 (3.1) | 45 (2.2) | 61 (3.0) | 63 (3.1) | 82 (4.2) | 0.003 |
| **Dyslipidemia medications** | 291 (3.7) | 53 (2.6) | 63 (3.2) | 71 (3.6) | 104 (5.4) | 0.000 |

**Table S1. Basic characteristics of participants**

BMI, body mass index; SBP, systolic blood pressure; DBP, diastolic blood pressure; TG, triglycerides; LDL-C, low-density lipoprotein cholesterol.

|  | **Events (%)** | **Crude model**  **OR (95% CI)** | **Adjusted model 1**  **OR (95% CI)** | **Adjusted model 2 OR (95% CI)** |
| --- | --- | --- | --- | --- |
| **MACE** |  |  |  |  |
| Quartile 1 | 311 (14.8) | reference | reference | reference |
| Quartile 2 | 340 (16.8) | 1.16(0.98-1.38) | 1.09(0.92-1.29) | 1.08(0.91-1.28) |
| Quartile 3 | 323 (15.7) | 1.07(0.90-1.27) | 0.99(0.83-1.17) | 0.97(0.82-1.16) |
| Quartile 4 | 414 (21.0) | 1.53(1.30-1.79) | 1.36(1.15-1.60) | 1.35(1.14-1.60) |
| **CVD** |  |  |  |  |
| Quartile 1 | 225 (10.7) | reference | reference | reference |
| Quartile 2 | 257 (12.7) | 1.21(1.00-1.47) | 1.13(0.93-1.37) | 1.12(0.92-1.36) |
| Quartile 3 | 216 (10.5) | 0.98(0.80-1.19) | 0.91(0.74-1.11) | 0.90(0.74-1.10) |
| Quartile 4 | 328 (16.6) | 1.66(1.38-1.99) | 1.47(1.22-1.77) | 1.48(1.22-1.78) |
| **Stroke** |  |  |  |  |
| Quartile 1 | 109 (5.2) | reference | reference | reference |
| Quartile 2 | 106 (5.3) | 1.01(0.77-1.33) | 0.95(0.72-1.25) | 0.93(0.70-1.23) |
| Quartile 3 | 132 (6.4) | 1.25(0.96-1.63) | 1.13(0.87-1.48) | 1.10(0.84-1.44) |
| Quartile 4 | 130 (6.6) | 1.29(0.99-1.67) | 1.13(0.87-1.48) | 1.11(0.85-1.45) |

**Table S2. Odds ratio with 95% confidence interval from univariate and multivariable models regarding the association between PM2.5 and cardiovascular outcomes**

Adjusted model 1 included age, gender, education, residence, body mass index, smoking status, alcohol consumption at baseline;Adjusted model 2 included model 1 plus hypertension status, diabetes status, dyslipidemia status, kidney disease status at baseline;Quartile 1 was used as the reference. Major adverse cardiovascular event incidence refers to the first ever cardiovascular disease or stroke event. MACE, major adverse cardiovascular event; CVD, cardiovascular disease; OR, odds ratio; CI, confidence interval.

| Sampling time | Bacterial concentrations of bioaerosol |
| --- | --- |
| 9:00-12:00 am | 224 CFU/m^3^ |
| 14:00-17:00 pm | 749 CFU/m^3^ |

**Table S3. Bacterial concentrations of bioaerosol from garbage stations**

**
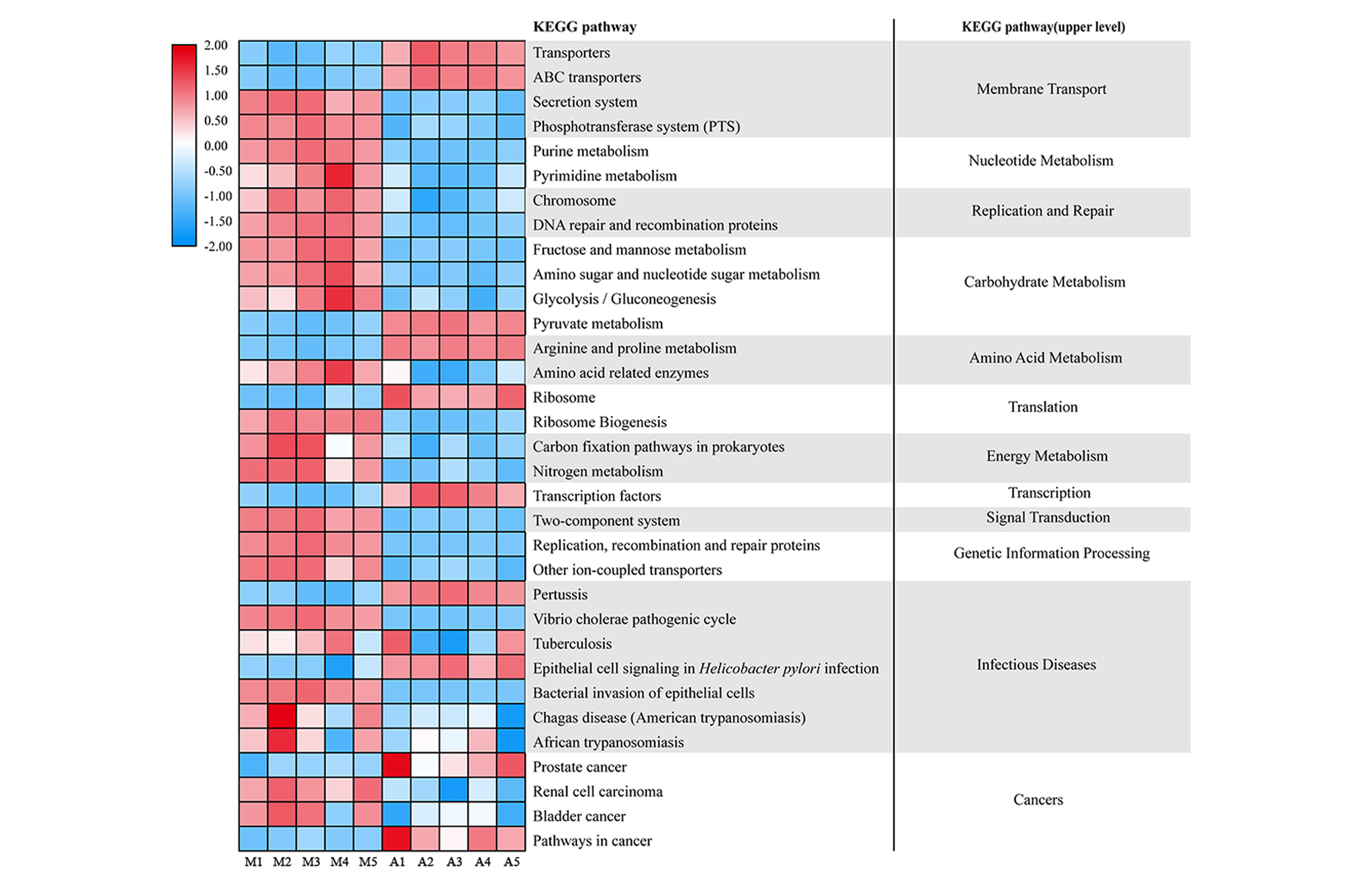
**

**Figure S1. KEGG genome functional comparative analysis in bioaerosols from garbage stations.**


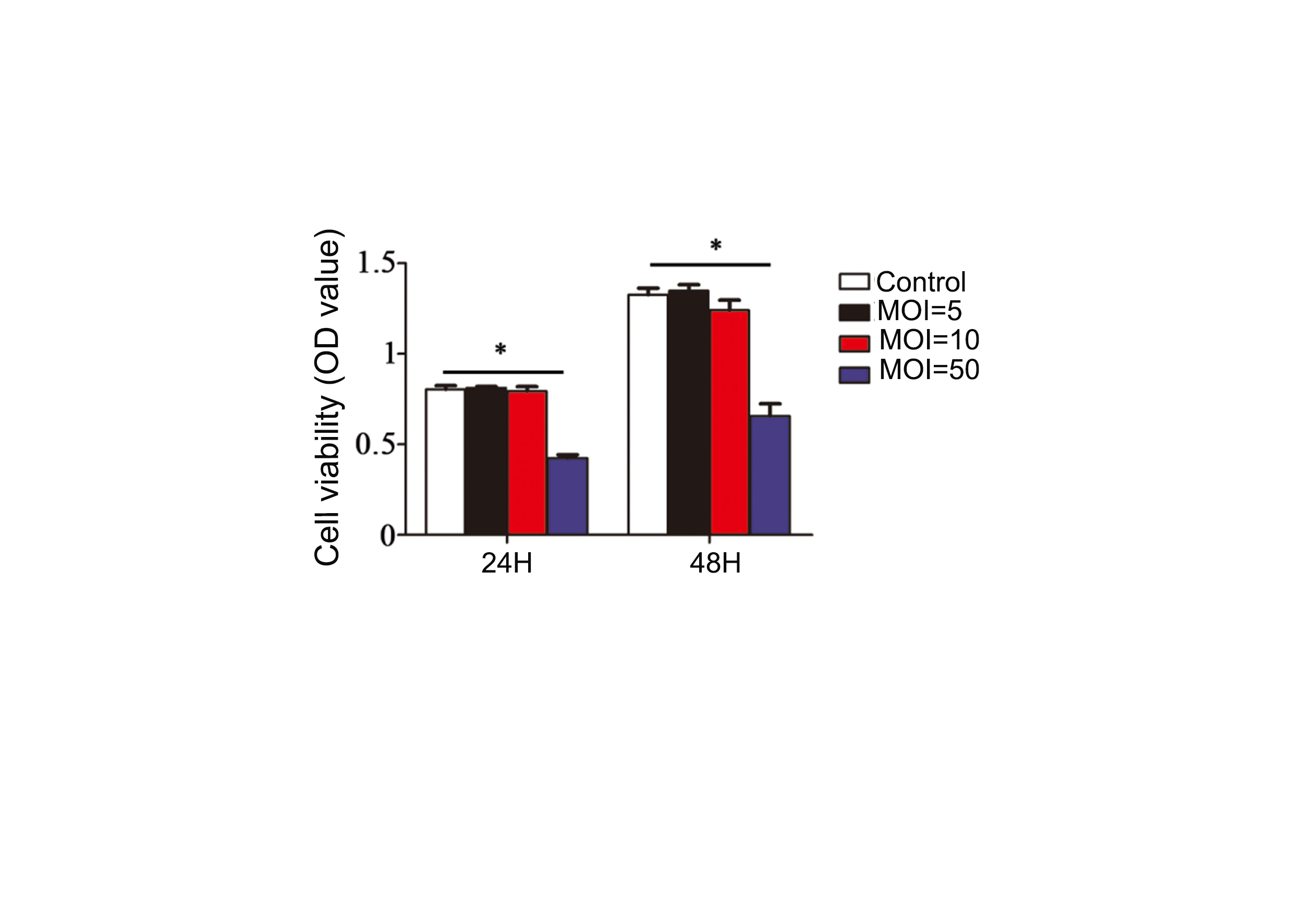


**Figure S2. Effects of bioaerosol on viability in HUVECs.**

HUVECs were treated with different concentrations of bioaerosol (MOI=5, MOI=10, MOI=50) for 24H and 48H, and cell viability was subsequently assessed. HUVECs：human umbilical vein endothelial cells. *p<0.05.


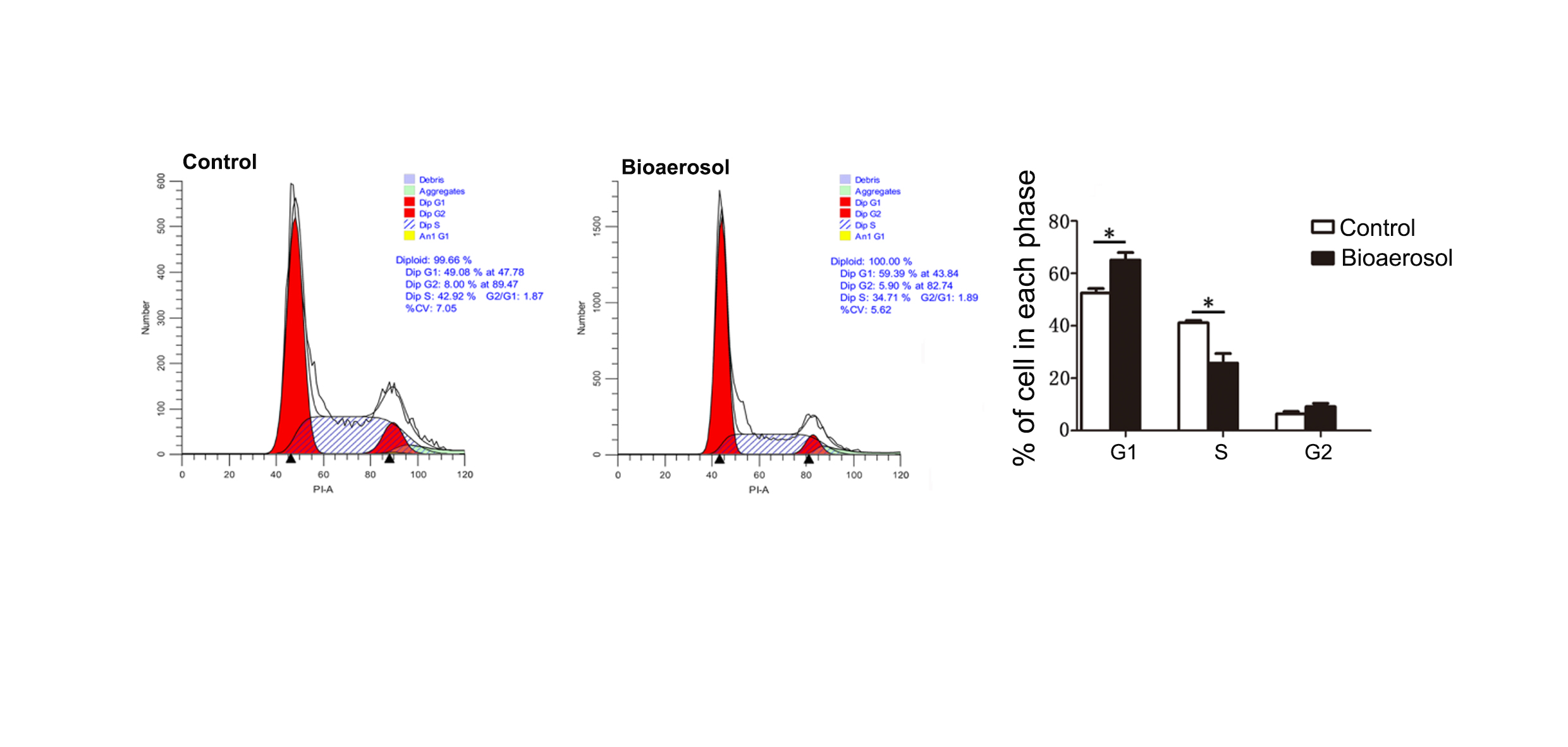


**Figure S3. Effects of bioaerosol on cell cycle in HUVECs.**

HUVECs were treated with bioaerosol (MOI=10) in the complete medium without antibiotics for 48 hours, and the effect of bioaerosol on the cell cycle was observed. *p<0.05.


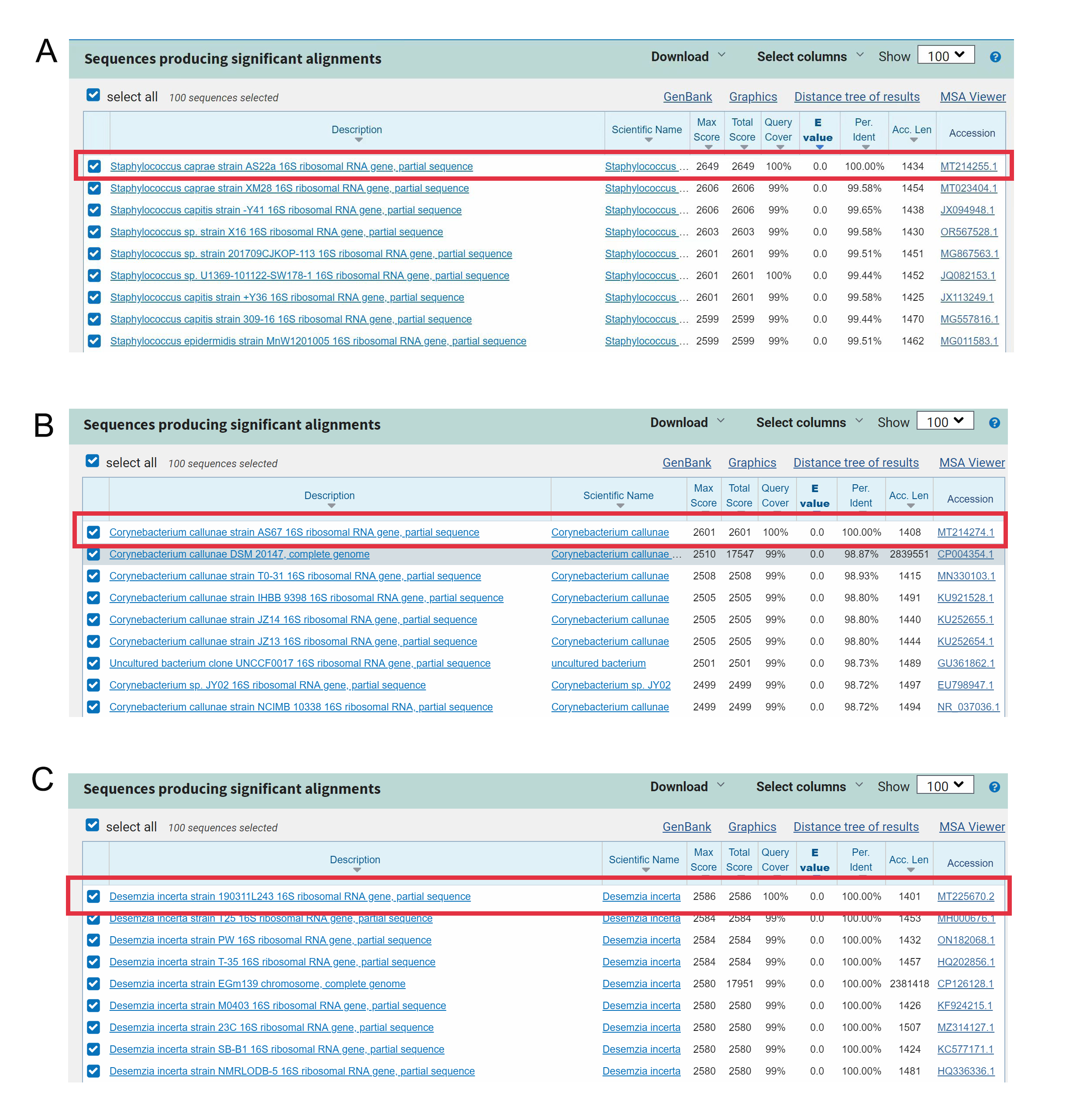


**Figure S4. Identification of bacteria in bioaerosol.**

After the isolated and purified strains were cultivated for several generations, the whole genome DNA was extracted, sequenced by 16SrDNA and Blasted, and the results were uploaded to the NCBI database. (A) AS22a Staphylococcus; (B) AS67 Corynebacterium; (C) L243 Desemzia.


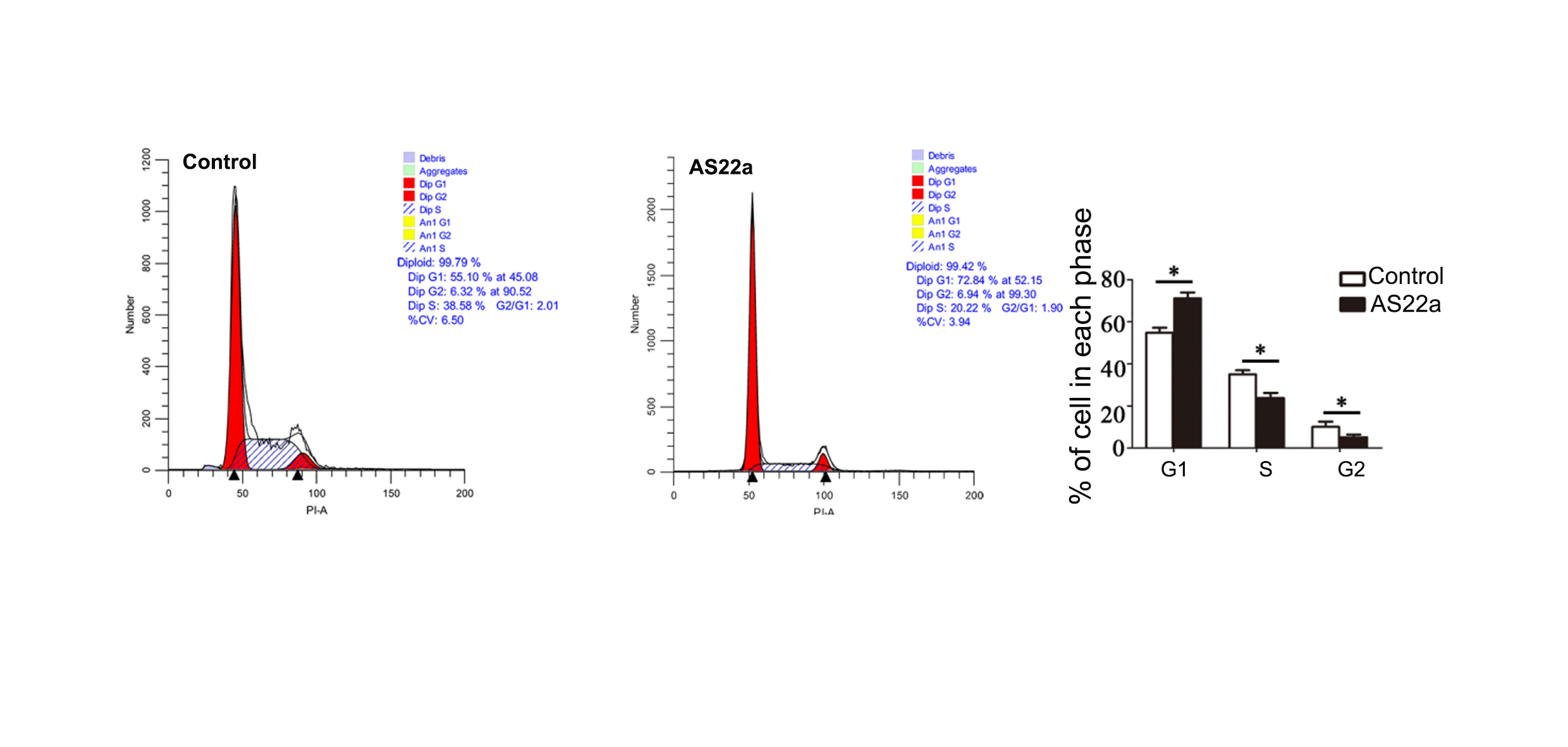


**Figure S5. Effects of AS22a on cell cycle in HUVECs.**

HUVECs were treated with AS22a (MOI=5) in the complete medium without antibiotics, and the effect of AS22a on the cell cycle was observed. *p<0.05.


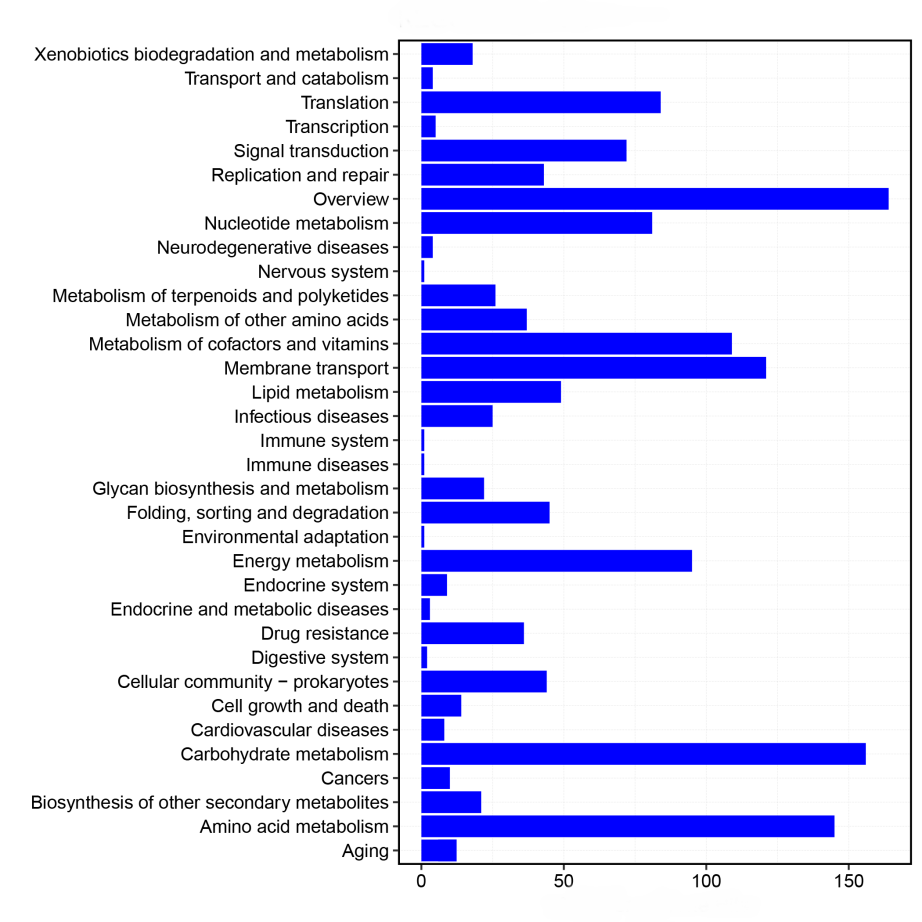


**Figure S6. The possible related functions of AS22a genome in HUVECs were predicted by 16SrRNA sequencing analysis.**
